# Validation of intra- and inter-laboratory reproducibility of the Xpert HPV assay according to the international guidelines for cervical cancer screening

**DOI:** 10.1186/s12985-018-1076-6

**Published:** 2018-10-29

**Authors:** Ajmal Akbari, Davy Vanden Broeck, Ina Benoy, Elizaveta Padalko, Johannes Bogers, Marc Arbyn

**Affiliations:** 1 AMBIOR, Laboratory for Cell Biology & Histology, University of Antwerp, Antwerp, Belgium; 2 National Reference Centre for HPV, Brussels, Belgium; 3 International Centre for Reproductive Health, Ghent University, Ghent, Belgium; 4 Laboratory of Molecular Pathology AML, Antwerp, Belgium; 5 Department of Microbiology and Immunology, Ghent University Hospital, Ghent, Belgium; 6 Unit of Cancer Epidemiology & Belgian Cancer Centre, Sciensano, Brussels, Belgium

**Keywords:** Human papillomavirus, Real-time sequencing, Cervical cancer screening

## Abstract

**Background:**

Cervical cancer screening with assays detecting DNA of high-risk human papillomavirus (hrHPV) types is more effective than cytology-based screening. This study completes the diagnostic accuracy assessment conducted previously within the framework of VALGENT-2 (Validation of HPV genotyping Tests) and aims to determine whether the reproducibility of Xpert HPV is in line with international validation criteria.

**Methods:**

Validation of new hrHPV DNA assays requires demonstration of good reproducibility and non-inferior clinical accuracy for cervical precancer compared to a standard comparator assay. The international reproducibility criteria are: lower bound of 95% confidence interval of the intra- and inter-laboratory agreement regarding detection of high-risk HPV DNA exceeding 87% with kappa ≥0.5.

**Results:**

The Xpert HPV assay showed high intra-laboratory reproducibility with an overall positivity/negativity agreement of 96.9% and a kappa of 0.925. Inter-laboratory testing showed an agreement of 97.8% with a kappa of 0.948.

**Conclusions:**

The Xpert HPV assay fulfills the HPV test reproducibility criterion requirement for use in cervical cancer screening.

## Background

High level of evidence from large randomized trials currently exists which indicates that human papillomavirus (HPV) based assays are more effective than cytology screening to reduce the burden of severe cervical dysplasia and cancer [1]. Two HPV assays were used in the randomized trials, Hybrid Capture 2 (HC2) and GP5+/6+ polymerase chain reaction-enzyme immunoassay (PCR-EIA).

International guidelines have been widely adopted for the clinical validation of novel HPV tests. In a population of screened women of at least 30 years of age, the novel HPV test should demonstrate non-inferior sensitivity and specificity for cervical intraepithelial neoplasia grade 2+ (CIN2+) compared to the standard comparator tests (Hybrid Capture 2 (HC2) HPV DNA test or GP5+/6+ PCR EIA) [2]. In addition, the novel HPV test should demonstrate high intra- and inter-laboratory reproducibility with a lower confidence bound not less than 87% while maintaining a kappa value higher than or equal to 0.5.

Based on meta-analysis, a list was established of high-risk HPV (hrHPV) assays that fulfill the cross-sectional performance criteria for cervical cancer screening [3]. This list is continuously updated as soon as data on new HPV assays become available. This current study aims to assess the intra- and inter-laboratory reproducibility of the Xpert HPV assay.

Xpert HPV is a non-batch real-time PCR assay capable of detecting 14 hrHPV types (HPV16, − 18, − 31, − 33, − 35, − 39, − 45, − 51, − 52, − 56 -58, − 59, − 66, − 68) within the run time of 1 h. The assay utilizes six fluorescent channels for the detection of individual types of hrHPV, groups of hrHPV, and the human reference gene. Fluorescent channel 1 detects HPV 16; fluorescent channel 2 detects HPV 18 and − 45; fluorescent channel 3 detects HPV 31, − 33, − 35, − 52, − 58; fluorescent channel 4 detects HPV 51, − 59; fluorescent channel 5 detects HPV 39, − 56, − 66, − 68 and fluorescent channel 6 is used for Sample Adequacy Control (SAC).

The fulfillment of the international equivalency criteria of Xpert HPV regarding sensitivity and specificity to detect cervical precancer has been evaluated as part of a separate study (VALGENT) [7, 9].

## Methods

VALGENT is an established comprehensive study design allowing validation of high-risk HPV DNA assays which potentially can be used for primary cervical cancer screening [2, 9]. It includes comparing the sensitivity and specificity for CIN2+ with a standard comparator test. Non inferior accuracy of the Xpert HPV assay compared to the GP5+/6+ PCR-assay was demonstrated in the second installment of VALGENT using samples collected from women attending the Scottish cervical cancer screening program [9]. The current study address the intra- and inter-reproducibility of the Xpert HPV assay using residual material of cervical specimens remnant after HPV testing with the AML qPCR and microscopic cytological processing. The cervical samples were collected in PreservCyt liquid based cytology media (ThinPrep**®**) (Hologic, San Diego, CA, USA) according to the manufacturers recommendations in agreement with European guidelines. Samples were collected between February 2016 and November 2016. Three aliquots of 1 ml of the original Preservcyt solution were prepared and stored at room temperature. The time span between specimen collection and preparation was maximum 3 days. The first two were tested independently with the Xpert HPV assay of the department of Cytopathology and Molecular Diagnostics at Algemeen Medisch Laboratorium (AML) with an interval of 4 weeks. A third aliquot was sent to laboratory of the Department of Microbiology and Immunology, Ghent University Hospital, Ghent, Belgium where it was tested again with the Xpert HPV assay.

The study set contained residual cervical cell material collected from 510 women attending cervical cancer screening in Belgium [10]. The composition was determined as recommended in the international guideline for validation of new hrHPV DNA tests [2], prescribing 30% of high-risk HPV positive specimen tested with a clinically validated assay. The study set contained aliquots form 357 hrHPV-negative and 153 hrHPV-positive women. HPV positivity was determined by the RIATOL real-time PCR targeting E6/7 genes of 18 HPV genotypes [4].

The RIATOL real-time qPCR allows genotyping of 18 HPV types for 14 hrHPV types (HPV 16, 18, 31, 33, 35, 39, 45, 51, 52, 56, 58, 59, 66, and 68), one probable hrHPV type (HPV 53), two low-risk types (HPV 6, 11) and one undetermined risk type (HPV 67). The AML HPV test has been clinically validated according to the international guidelines [5]. A detailed description of the laboratory developed validation test has been published previously. The RIATOL real-time qPCR generates a quantitative correlation by generating signal strength in the form of Ct-values (cycle threshold). The Ct-values are calculated by determining the point at which the fluorescence exceeds a threshold limit [6]. Ct-values are inversely proportional to the amount of target nucleic acid in a sample. Studies have shown that AML HPV test is highly sensitive for the detection of HPV genotypes [7].

The overall percentage of agreement was computed as the proportion of concordant results (positive + negative concordant test results) over all test results with corresponding binomial exact 95% confidence intervals. Cohen’s Kappa values were calculated and 95% CI as proposed by Fleiss [8]. Intra-reproducibility assessment was based on the two Xpert HPV testings in the AML laboratory. Inter-reproducibility assessment was based on the first testing in AML and the testing at the University of Gent. The reproducibility validation criterion for new hrHPV DNA assays usable in cervical screening was considered as fulfilled when the left 95% confidence interval (CI) bound for hrHPV concordance exceeded 87% and the kappa > 0.5.

The Ct-values per fluorescent channel were recorded during each run. To identify the consistency of these values during the intra- and inter-laboratory tests, the average, minimal and maximal difference in Ct-values of the samples were calculated per fluorescent channel.

## Results

The results from hrHPV testing with Xpert HPV showed high intra-laboratory reproducibility with an agreement of 96.9% (95% CI, 95.0–98.2%) (494/510 samples) and a kappa of 0.925 (95% CI 0.888–961). The inter-laboratory test showed an excellent agreement of 97.8% (95% CI, 96.2–98.9%), 499/510 samples) with a kappa of 0.948 (95% CI 0.917–0.978). The results of intra- and inter-laboratory reproducibility are represented in Table 1.

**Table 1:** Intra and inter laboratory reproducibility

Intra-laboratory reproducibility^a^			
	Xpert HPV – AML Run 2		Total
Xpert HPV –AML Run 1	Negative	Positive	
Negative	353	4	357
Positive	12	141	153
Total	365	145	510
Inter-laboratory agreement^b^			
	Xpert HPV UZ Ghent		Total
Xpert HPV –AML Run 1	Negative	Positive	
Negative	356	1	357
Positive	10	143	153
Total	366	144	510
Inter-laboratory agreement^c^			
	Xpert HPV UZ Ghent		Total
Xpert HPV –AML Run 2	Negative	Positive	
Negative	154	3	357
Positive	4	149	153
Total	157	153	510

^a^The overall HPV test agreement 96.9% (95% CI, 95.0–98.2%) KAPPA value of 0.925 (95% CI 0.888–0.961)

^b^The overall HPV test agreement 97.8% (95% CI, 96.2–98.9%) KAPPA value of 0.948 (95% CI 0.917–0.978)

^c^The overall HPV test agreement 98.6% (95% CI, 97.6–99.6%) KAPPA value of 0.967 (95% CI 0.943–0.991)

Results were evaluated per fluorescent channel. The overall agreement within the intra-laboratory runs was close to 100%, with the lower confidence bound not less than 96%. The lowest overall agreement of 98% was detected in fluorescent channel 3 (HPV 31, 33, 35, 52, and 58) between the intra-laboratory run 1 and run 2. Other runs showed that the agreement within fluorescent channel 3 was slightly lower. The kappa value of the intra-laboratory reproducibility run was close to 1.

There were inconsistencies noticed in the Ct- values annotated to the sample within the three Xpert HPV runs. Table 2 represents the summary of the samples with inconsistent Ct-values in the five fluorescent channels during the intra-and inter-laboratory tests. The average difference within the Ct-values of the five fluorescent channels was 1.1 and varied between 0.1 and 3.2. The largest difference in Ct-values (MAX 8.6, 6.7, and 8.4) can be observed in fluorescent channel 3 (HPV type 31, 33, 35, 52, and 58) for the three runs, respectively.

**Table 2:** Difference in Ct-values between intra- and inter- laboratory runs

	N	AVG	Range	
			MIN	MAX
AML run 1 versus AML run 2				
FC 1	30	1.1	0.1	3.2
FC 2	22	0.9	0.1	3.8
FC 3	64	1.4	0.1	8.6
FC 4	29	1.0	0.1	3.7
FC 5	42	1.3	0.2	4.8
AML run 1 versus Ghent run				
FC 1	31	1.2	0.1	3.3
FC 2	21	1.3	0.1	3.9
FC 3	63	1.3	0.1	6.7
FC 4	29	1.3	0.1	7.1
FC 5	40	1.3	0.1	4.2
AML run 2 versus Ghent run				
FC 1	27	1.2	0.1	3.7
FC 2	23	1.2	0.1	5.0
FC 3	65	1.2	0.1	8.4
FC 4	29	1.2	0.1	6.1
FC 5	43	1.3	0.1	6.0

*FC* Fluorescent channel, *N* Number of samples, *AVG* Average, *MAX* maximum, *MIN* minimum

The overall agreement within the inter-laboratory runs was close to 100%, with the lower confidence bound not less than 97% while a slightly lower agreement was found for fluorescent channel 3. The kappa value of the inter-laboratory reproducibility run was close to 1. The reproducibility of the Xpert HPV per fluorescent channel is represented in Table 3.

**Table 3:** Overall agreement, lower and upper confidence bound, and the KAPPA value of each flourescent channel per run

	Agreement	95% CI		Kappa	Kappa 95% CI	
		Lower bound	Upper bound		Lower bound	Upper bound
Intra-laboratory run 1versusIntra-laboratory run 2						
Fluorescent channel 1	99.4%	98.8%	100%	0.99	97.0%	100%
Fluorescent channel 2	99.6%	99.1%	100%	0.99	97.8%	100%
Fluorescent channel 3	98.0%	96.8%	99.2%	0.95	92.4%	98.2%
Fluorescent channel 4	99.2%	98.5%	100%	0.98	96.3%	100%
Fluorescent channel 5	98.8%	97.9%	99.8%	0.97	95.0%	99.4%
Intra-laboratory run 1 versusInter-laboratory (Ghent)						
Fluorescent channel 1	99.6%	99.1%	100%	0.99	97.8%	100%
Fluorescent channel 2	99.6%	99.1%	100%	0.99	97.8%	100%
Fluorescent channel 3	98.8%	97.9%	99.8%	0.97	94.9%	99.4%
Fluorescent channel 4	99.2%	98.5%	100%	0.98	96.3%	100%
Fluorescent channel 5	98.6%	97.6%	99.6%	0.97	94.3%	99.1%
Intra-laboratory run 2 versusInter-laboratory (Ghent)						
Fluorescent channel 1	99.4%	98.8%	100%	0.99	97.0%	100%
Fluorescent channel 2	100%	100%	100%	1.00	100%	100%
Fluorescent channel 3	98.8%	97.9%	99.8%	0.97	95.0%	99.4%
Fluorescent channel 4	99.2%	98.5%	100%	0.98	96.3%	100%
Fluorescent channel 5	99.8%	99.4%	100%	1.00	98.6%	10%

*CI* Confidence interval, *Fluorescent channel FC 1* detects HPV16, *FC2* detects HPV18 and HPV45, *FC3* detects HPV31,33,35,52,58, *FC4* detects HPV51,59, *FC5* detects HPV 39,56,66,68

## Discussion and conclusions

During the intra- and inter- laboratory reproducibility testing 1530 Xpert HPV assay runs were conducted. Sixteen samples that were identified as INVALID by the Xpert HPV assay. These samples were re-tested and the results of the assay turned out to be valid after re-testing. This implies that the internal control at the initial testing was indicating assay failure rather than sample failure. Xpert HPV has shown excellent reproducibility but it also has an error rate of approximately 1%. An error rate of 1% can be significant when large numbers of samples are analyzed with Xpert HPV.

Xpert HPV showed excellent overall intra-laboratory reproducibility with an agreement of 96.9% and a kappa of 0.925). The overall reproducibility was close to 100% for HPV 16 and HPV 18/45, confirming reliability of Xpert HPV for the main high-risk types. Besides that the inter-laboratory test showed an excellent agreement of 97.8% with a kappa of 0.948 and great reproducibility statistics for HPV16 and HPV18/45. A small number of discrepancies were found, but almost all of them appeared to be linked to detection limit, Ct-value or borderline issues.

Our data confirm that the intra-laboratory reproducibility and inter-laboratory agreement of Xpert HPV largely exceeds the requirements formulated in the international guidelines for the validation of a novel hrHPV test with respect to its use in primary cervical cancer screening. Our study completes the VALGENT-2 clinical validation study which demonstrated a relative sensitivity and relative specificity of hrHPV testing with Xpert HPV compared to GP5+/6+ PCR of 0.984 (95% CI 0.931–1.040, p_non-inferiority_ [p_ni_] = 0.019) and 1.006 (95% CI 0.997–1.016, p_ni_ < 0.0001), respectively, among screened women aged 30 or older [9]. Considering the VALGENT findings with the reproducibility results from the current study, we can conclude that the Xpert HPV can be added to the list of assays that fulfill international criteria for primary cervical cancer screening [3]. In addition, Xpert HPV is hereby the only point of care test fulfilling these criteria.

## Abbreviations

CIN2+: Cervical intraepithelial neoplasia grade 2+
Ct: Cycle threshold
DNA: Deoxyriboynucleic acid
EIA: Enzyme immunoassay
HC2: Hybrid capture 2
HPV: Human papillomavirus
HrHPV: High risk human papillomavirus
PCR: Polymerase chain reaction
SAC: Sample adequacy control
